# Biotic Potential Induced by Different Host Plants in the Fall Armyworm, *Spodoptera frugiperda* (Lepidoptera: Noctuidae)

**DOI:** 10.3390/insects13100921

**Published:** 2022-10-12

**Authors:** Nimra Altaf, Atif Idrees, Muhammad Irfan Ullah, Muhammad Arshad, Ayesha Afzal, Muhammad Afzal, Muhammad Rizwan, Jun Li

**Affiliations:** 1 Department of Entomology, University of Sargodha, Sargodha 40100, Pakistan; 2 Guangdong Key Laboratory of Animal Conservation and Resource Utilization, Guangdong Public Laboratory of Wild Animal Conservation and Utilization, Institute of Zoology, Guangdong Academy of Sciences, Guangzhou 510260, China; 3 Institute of Molecular Biology and Biotechnology, The University of Lahore, 1-Km Defense Road, Lahore 54000, Pakistan; 4 Beekeeping and Hill Fruit Pests Research Station, Rawalpindi 46300, Pakistan

**Keywords:** life table, *Spodoptera frugiperda*, insect-plant interaction, fecundity, population parameters

## Abstract

**Simple Summary:**

Fall armyworm, *Spodoptera frugiperda* (Lepidoptera: Noctuidae), is an economically important insect pest of corn crops globally. However, some other host plants on which this pest can successfully complete its generation have also been reported. Our main objective was to study the biology of fall armyworm feeding on maize, sorghum, wheat, and rice. Our overall findings show that maize is the most preferred host plant; however, the pest completed the life cycle successfully on sorghum and wheat. The survival rate was low when rice leaves were provided to larvae as diet, suggesting that rice is a non-preferred host plant.

**Abstract:**

Fall armyworm, *Spodoptera frugiperda* (J.E. Smith) (Lepidoptera: Noctuidae), is a polyphagous insect pest of many important crops. To evaluate the influence of host plants on the biology and survival of the Pakistani population of *S. frugiperda*, we examined life table parameters of *S. frugiperda* raised on maize, sorghum, wheat, and rice. The development rate was significantly higher on the maize crop than on the other three host plants. Different larval diets affected development time and fecundity. *S. frugiperda* attained the fastest larval development (16 days) on maize and the slowest development (32.74 days) on rice. Adult females from maize-fed larvae laid 1088 eggs/female, those from sorghum-fed larvae laid 591.6 eggs/female, those from wheat-fed larvae laid 435.6 eggs/female, and those from rice-fed larvae laid 49.6 eggs/female. Age stage-specific parameters also indicated the higher fecundity, higher life expectancy, and higher survival of *S. frugiperda* on maize plants than on the other three hosts. Larval diets had a significant varying effect on the finite and intrinsic increase rates, reflecting that maize was the most suitable diet. The findings of the present study are useful for predicting population dynamics especially in areas cultivating Poaceae crops, except maize, to develop sustainable integrated pest management strategies for this pest.

## 1. Introduction

Fall armyworm, *Spodoptera frugiperda* (J.E. Smith) (Lepidoptera: Noctuidae), is a polyphagous pest that originated from the American continents. It feeds on approximately 353 plant species belonging to 76 plant families and prefers to feed on economically important crops such as maize, sorghum, rice, millet, and sugarcane [1,2,3,4,5,6,7]. *S. frugiperda* has the ability to damage various crops rapidly and hence deteriorates the nutritional value of the infested crops [8]. This pest has spread into all of northeastern India and damaged the maize crop [9]. Before 2016, *S. frugiperda* was only found in South and North America. The occurrence of this pest was reported in Africa in 2016 [10] and spread in Europe in 2018 [11]. In Asia, it was first reported in India in 2018 [12] and damaged the maize crop [9]. A year after the first invasion into Asia, *S. frugiperda* was found in Indonesia and West Africa [13,14]. In Pakistan, *S. frugiperda* was initially found on maize crop in the Sindh province in the southern part of Pakistan in 2019, and has now spread to different regions of the country and affects maize, millet, and sorghum [15,16,17,18]. The damage amount of *S. frugiperda* feeding on maize crop is substantial; losses of 73% in Latin America [19] and 21–53% in Africa [2] have been reported.

*S. frugiperda* larvae feed on the stem, leaves, and reproductive parts of their host plants [20]. Two strains of *S. frugiperda* have been reported worldwide: corn strain and rice strain. The corn strain mostly prefers maize and sorghum, while the rice strain mostly prefers pastures including rice [21,22]. The change in the population of any insect pest depends on the nutrition and properties of their host plants, which influence their population growth [23,24]. Life history traits of insects, including growth, reproduction, survival, etc., are affected by the different nutrition of different host plants that insects feed on during their larval stages. Demographic studies play an important role in population dynamics and pest status in the field [25,26]. Although the most preferable crop of *S. frugiperda* is maize [27], other crops can be suitable hosts in the absence of maize crops. Given the further dispersion of *S. frugiperda* in Pakistan, there is a dire need to reveal the biological performance of this pest on other economically important crops such as wheat, sorghum, and rice.

*S. frugiperda* very recently invaded Asian countries, including China [28]. It was recorded for the first time in Pakistan in 2019 [17,29]. Therefore, little information on the developmental biology, biotic potential, and life history parameters of this novel pest feeding on diverse plant species is available in Pakistan. Thus, there is a need to conduct a comprehensive study on the biology and life history of *S. frugiperda* on various host plants. To understand and predict the population growth, life table theory gives analyses for the development and fecundity of the next generation [30] and it is helpful to study the biology of insect populations and community ecology analyses. It helps to distinguish different life stages and comprises both sexes in analyzing, interpreting, and explaining recorded data [31]. We aimed to evaluate the impact of four host plants, namely maize, wheat, sorghum, and rice, on the life table parameters of *S. frugiperda*. As the maize crop is the most preferred host plant for the corn strain of *S. frugiperda*, the biotic potential of this pest was tested on wheat, sorghum, and rice compared to maize.

## 2. Materials and Methods

### 2.1. Host Plants Seedlings

Four host plants belonging to the family Poaceae, namely maize (*Zea mays* L.; var. HY-CORN 11 Plus), wheat (*Triticum aestivium* L.; var. Akbar 19), rice (*Oryza sativa* L.; var. Super Basmati), and sorghum (*Sorghum bicolor* L.; var. Sorgosweet), were selected based on their economic importance. Seeds were purchased from a local market in Sargodha, Pakistan. Seeds were sown in pots and kept in a greenhouse. Fresh and fully expanded leaves of plants were used as the diet for *S. frugiperda* larvae.

### 2.2. Rearing Colony of S. frugiperda

The larvae of *S. frugiperda* were collected from a maize field in the Sargodha district. The eggs and larvae were reared in the Entomology Laboratory at the University of Sargodha. Neonate larvae were reared on an artificial diet. The artificial diet was prepared according to the method suggested by [32,33]. After the emergence of adult moths, pairs of male and female moths were confined to oviposition jars. Adults were fed 10% sugar solution. Muslin cloth was provided in plastic pots to facilitate the oviposition. The culture was maintained at 60–70% relative humidity and 25 ± 1 °C temperature in the laboratory. The insects were reared for three consecutive generations in the laboratory.

### 2.3. Life Table Studies

Five egg masses laid within 6 h were randomly collected from the reared colony and kept in clean Petri plates. One-day-old newly hatched first instar larvae were obtained and transferred to new Petri plates. For each host plant, 80 larvae were separated from the rearing colony and kept in Petri plates (one larva in each plate). Each larva was considered as one replication, totaling 80 replications for each treatment. Fresh leaves of each host plant were supplied as needed until pupation. The development and survival of larvae were checked daily. After pupal formation, all pupae were shifted into plastic cups lined with cotton and were monitored daily. After the pupae emerged as adults, males and females were paired and each pair was placed into transparent plastic boxes (25 cm × 25 cm × 25 cm). A single plant of maize and sorghum and almost 5 seedlings of wheat and rice, which had been planted in a disposable cup, were added to each of the plastic boxes as an oviposition substrate. Each oviposition cage also contained a small cotton ball soaked with 10% honey solution for adult feeding. A muslin cloth was also hung in plastic boxes to facilitate egg laying. New egg batches were collected and transferred to Petri plates, and the fecundity rate was recorded. This experiment was performed under controlled conditions at 25 ± 1 °C, 60–70% relative humidity, and a 16:8 h (light:dark, L:D) photoperiod. The duration of each stage of insect was recorded from egg incubation to adult life. From the same treatment, newly emerged male and female adults were paired and released into a separate cage to record the fecundity rate.

### 2.4. Life Table Analyses

We analyzed the raw data of development duration and survival by using age stage two-sex life table procedures and calculated the parameters of life table theory by using the computer program TWOSEX-MS Chart [31]. The bootstrapping method (with 100,000 random samplings) was used to calculate the standard error for the population in MS Chart program.

## 3. Results

The development of each stage of *S. frugiperda* on four plant species is given in Table 1. No significant difference in the duration of egg stage (*p > 0.05*) was found among the four populations whose larvae were provided different host plant species. However, the duration of all instars in larvae reared on maize, sorghum, and wheat was shorter compared to larvae reared on rice. The pupal duration was 9.0 ± 0.05_d on maize, 10.6 ± 0.12 d on sorghum, 11.8 ± 0.23 d on wheat, and 19.6 ± 0.72 d on rice. The mean longevity of adults reared on rice was 14.8 ± 0.26 d, which is shorter than 18.2 ± 0.17 d on maize, 18.9 ± 0.10 d on sorghum, and 22.2 ± 0.09 d on wheat (Table 1).

All the reproductive and life table parameters of *S. frugiperda* were significantly different (*p* < 0.05) on the four host plants. A shorter adult pre-oviposition period (*APOP*) was recorded on maize (3.07 ± 0.11 d) and sorghum (3.39 ± 0.10 d) than on wheat (4.92 ± 0.16 d) and rice (4.17 ± 0.32 d). The adult total pre-oviposition period (*TPOP*) was also shorter on maize and sorghum compared to wheat and rice. The oviposition period was longer on maize compared to the other host plants: 4.56 ± 0.08 d on maize, 3.87 ± 0.07 d on sorghum, 3.56 ± 0.07 d on wheat, and 2.0 ± 0.01 d on rice. The fecundity rate on maize (1088.8 ± 26.5 eggs/female) was higher compared to that on sorghum (591.6 ± 11.5 eggs/female), wheat (435.6 ± 6.91 eggs/female), and rice (49.6 ± 4.54 eggs/female). The net reproductive rate (*R*_0_) of *S. frugiperda* was higher on maize (735.1 ± 59.6 offspring) compared to sorghum (340.2 ± 33.3 offspring), wheat (272.09 ± 23.9 offspring), and rice (7.45 ± 2.08 offspring). Mean generation time (*T*) was recorded as 36.4 ± 0.182 d on maize, 41.5 ± 0.279 d on sorghum, 47.6 ± 0.37 d on wheat, and 61.9 ± 1.14 d on rice. Similarly, the intrinsic increase rate (*r*) and finite increase rate (*λ*) of larvae feeding on maize were higher than those of larvae on the other plants (Table 2).

**Figure 1:**
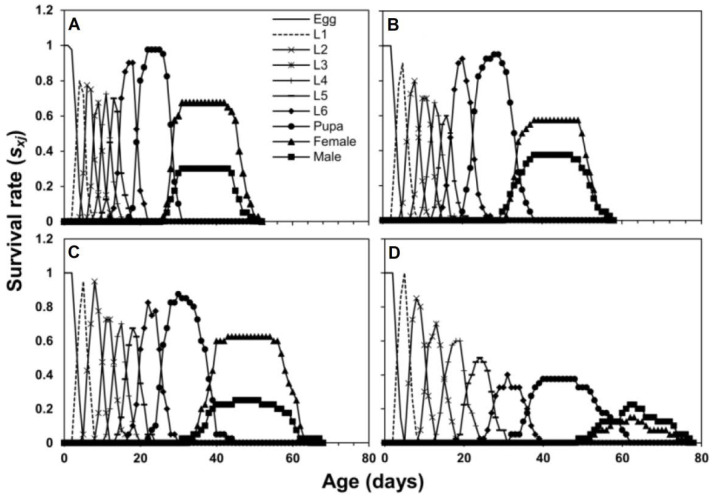
Age stage-specific survival rate (*s_xj_*) of *S. frugiperda* fed on four host plants, maize (**A**), sorghum (**B**), wheat (**C**) and rice (**D**).

**Figure 2:**
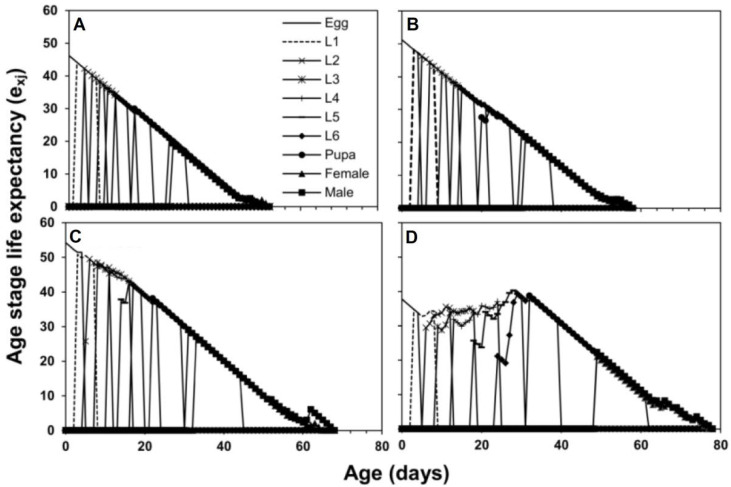
Age stage-specific life expectancy (*e_xj_*) of *S. frugiperda* fed on four host plants, maize (**A**), sorghum (**B**), wheat (**C**) and rice (**D**).

The age stage-specific survival rate (*s_xj_*) curves show that the survival rate of *S. frugiperda* was higher when they fed on maize compared to sorghum, wheat, and rice (Figure 1). The age stage-specific life expectancy (*e_xj_*) is given in Figure 2. Newly hatched larvae of *S. frugiperda* were predicted to live for 46.2 d on maize, 51.2 d on sorghum, 54.2 d on wheat, and 37.7 d on rice. The *e_xj_* of females was found to be greater on maize compared to males, while in the other three host plant treatments, the *e_xj_* of males was higher than females. The values of *e_xj_* for adult females were 21.1 d on maize, 21.5 d on sorghum, 27.1 d on wheat, and 21.0 d on rice. The values of *e_xj_* for adult males were 19.3 d on maize, 22.8 d on sorghum, 29.0 d on wheat, and 22.2 d on rice (Figure 2).

**Figure 3:**
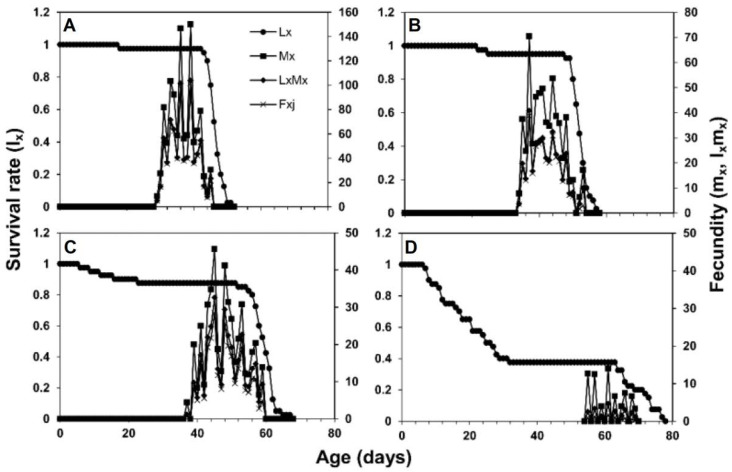
Age-specific survival rate (*l_x_*), age stage-specific fecundity (*f_xj_*), age-specific fecundity (*m_x_*), and age-specific maternity (*l_x_m_x_*) of *S. frugiperda* fed on four host plants, maize (**A**), sorghum (**B**), wheat (**C**) and rice (**D**).

**Figure 4:**
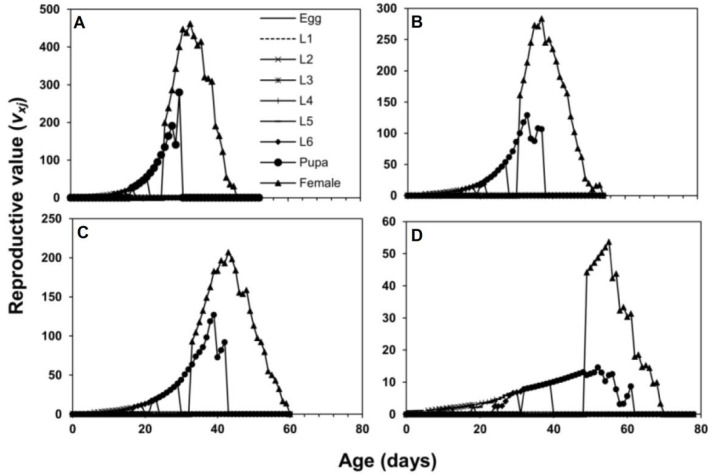
Age stage-specific reproductive rate (*v_xj_*) of *S. frugiperda* fed on four host plants, maize (**A**), sorghum (**B**), wheat (**C**) and rice (**D**).

The female fecundity (*f_xj_*) showed that 101.3 eggs on the 39th day, 38.7 eggs on the 37th day, 28.5 eggs on the 45th day, and 1.75 eggs on the 61st day were laid on maize, sorghum, wheat, and rice, respectively. The age-specific survival rate (*l_x_*) of *S. frugiperda* was higher on maize and sorghum than on wheat and rice (Figure 3). The values of age stage-specific reproductive rate (*v_xj_*) of an adult female were recorded with the following trend: 461.7 at the 33rd day on maize, 283.5 at the 37th day on sorghum, 206.9 at 43rd day on wheat, and 53.71 at 55th day on rice (Figure 4).

## 4. Discussion

Since 2019, when Naeem-Ullah and colleagues reported that *S. frugiperda* larvae feeding on maize were found for the first time in Pakistan [17], this pest has spread to almost all the maize-producing areas of Pakistan. Given the polyphagous nature of this larva, it could be a threat to other economically important crops such as sorghum, wheat, cotton, and rice. The varying nutritional values of crop species and variations have a significant impact on insect growth, development, and survival [3,34]. This study revealed the impact of four host plants (diets) on the development and survival of *S. frugiperda*. The results indicate that each larval instar developed faster while feeding on the maize plant; however, the growth on sorghum and wheat was comparable to that on maize, but the performance on rice was crucially low. All larval instars and pupae showed the shortest development duration on maize, and the longest development was recorded on rice. The highest survival of *S. frugiperda* was recorded on maize and the lowest survival was recorded on rice. Previous studies showed the shortest pupal duration on maize compared to potato and tobacco [35]. The higher survival rate and shorter life cycle of *S. frugiperda* on maize show that maize is one of the preferred hosts [35].

The nutrition of the larval diet has a significant impact on adult fecundity and adult duration, as well [36]. *S. frugiperda* fed on maize showed the highest fecundity and survival rate, whereas the fecundity rate of *S. frugiperda* fed on rice was low. Previous studies also showed the lowest pupal duration of *S. frugiperda* on maize [35]. Female from the larvae fed on maize leaves laid 1088 eggs and the average oviposition period was 4.56 days, and 591.6 eggs on sorghum and 435.6 eggs on wheat were recorded. In the case of rice, only 49.6 eggs were recorded, and the oviposition period was only 2 days. The net reproductive rate was higher on maize, followed by sorghum and wheat, and the lowest reproductive rate was recorded on rice. A more detailed study is needed to confirm how differences in the nutritional contents and defensive compounds among plants affect the development, survival, and fecundity relevant to population dynamics of insect pests.

The suitability of host plants can be assessed through different parameters such as larval development duration, fecundity, and mean generation time. The shorter developmental period and the high reproduction potential were achieved by feeding on maize plants, supporting that maize is suitable for *S. frugiperda* larval diet. This preference was also supported by the positive impact on the reproductive parameters, including *r*, λ, and *R_0_*. Values of these parameters were higher in the case of maize compared to the other three hosts. The life table parameters *r*, λ, *R_0_,* and *T* express the capacity of insect growth for a given population in a specific environment [36]. The lower survival rate, oviposition period, and fecundity rate and longer developmental time of *S. frugiperda* reared on sorghum, wheat, and rice resulted in lower *r*, λ, and *R_0_* values and higher *T* values as compared to maize. This might be due to the nutritional differences in the host plants. In this study, the higher fecundity of *S. frugiperda* reared on maize suggests that maize is a more susceptible host plant than sorghum, wheat, and rice. In addition, the longer oviposition duration, shorter APOP and TPOP, and higher population parameters (*r*, λ, and *R_0_*) indicate that maize is a more susceptible host than other plants.

The survival rate (*s_xj_*) of *S. frugiperda* was higher on maize compared to sorghum, wheat, and rice. Life expectancy (*e_xj_*) of males and females also varied on the four host plants. The *e_xj_* of females was greater on maize compared to males. These parameters are used to establish early warning models for predicting insect survival at a specific age and then pest occurrence timing and amount. This strategy is helpful in pest management programs [37,38]. Higher reproduction of female *S. frugiperda* on maize, sorghum, and wheat than on rice plants would drive a significant population growth on these crops in the field [39]. In the absence of maize, *S. frugiperda* may complete its life cycle on wheat [40] and sorghum [41]. Our study conducted under controlled conditions suggests that *S. frugiperda* highly preferred maize to sorghum, wheat (intermediate), and rice (least preferred). To demonstrate this preference and predict future population growth, a field study on the impact of these host plants on *S. frugiperda* is necessary. 

Goergen et al. [10] conducted a study on the performance of *S. frugiperda* on maize, potato, and tobacco crops. They reported the highest development performance of *S. frugiperda* on maize among the three crops. Wu et al. [42] reported that *S. frugiperda* larvae completed development on tomato and pepper but not on eggplant. Interestingly, *S. frugiperda* larvae fed on tomato plants had better fitness compared to those fed on maize [42]. In Asia, therefore, this pest possibly infests sorghum, wheat, and tomato, especially in the absence of a preferred host (maize). Even though the corn strain of *S*. *frugiperda* is closely associated with the corn crop, it can also damage sorghum, wheat, and rice. This pest can adapt to other crops in the absence of a preferred host, as reported earlier [43,44,45,46]. 

## 5. Conclusions

Our findings show that populations of *S. frugiperda* collected from maize had higher growth rates. However, in the absence of maize, sorghum and wheat were also suitable hosts for this pest and may serve as alternative hosts for the reproduction of S. *frugiperda*. Thus, sorghum and wheat crops may also face threats from *S. frugiperda* if grown in areas near maize. The results of this study are useful in predicting population dynamics, especially in areas cultivating Poaceae crops, and will aid in the development of sustainable integrated pest management strategies for *S. frugiperda*.

## Figures and Tables

**Table 1:** Development time (days) of (mean ± SE) *S. frugiperda* raised on different hosts.

Life Stage	*n*	Maize	*n*	Sorghum	*n*	Wheat	*n*	Rice
Egg	80	3.77 ± 0.09 a	80	3.88 ± 0.08 a	80	3.88 ± 0.09 a	80	3.70 ± 0.08 a
L1	80	2.17 ± 0.04 c	80	2.88 ± 0.07 b	80	2.90 ± 0.07 b	80	3.25 ± 0.05 a
L2	80	2.33 ± 0.06 d	80	2.88 ± 0.07 c	76	3.68 ± 0.05 b	70	4.11 ± 0.10 a
L3	80	2.10 ± 0.06 d	80	2.95 ± 0.08 c	74	3.19 ± 0.09 b	60	4.67 ± 0.08 a
L4	80	2.33 ± 0.05 d	80	2.67 ± 0.07 c	74	3.19 ± 0.05 b	52	5.69 ± 0.08 a
L5	80	2.45 ± 0.08 b	80	2.40 ± 0.06 c	72	3.44 ± 0.07 a	40	6.75 ± 0.10 a
L6	78	4.62 ± 0.06 d	80	5.42 ± 0.06 c	70	5.80 ± 0.05 b	30	8.27 ± 0.16 a
Pupa	78	9.00 ± 0.05 d	76	10.6 ± 0.12 c	70	11.8 ± 0.23 b	30	19.6 ± 0.72 a
Adult	78	18.2 ± 0.17 d	76	18.9 ± 0.10 c	70	22.2 ± 0.09 b	30	14.8 ± 0.26 a
Male	24	47.1 ± 0.27 d	30	52.8 ± 0.47 c	20	60.0 ± 0.91 b	18	71.2 ± 1.17 a
Female	54	46.3 ± 0.35 d	46	52.5 ± 0.28 c	50	60.1 ± 0.35 b	12	70.0 ± 1.40 a

*n* = number of individuals; means sharing similar letters within a row are not significantly different at *p* > 0.05; L1–L6 indicate larval instars.

**Table 2:** Comparison of reproductive and life table parameters (mean ± SE) of *S. frugiperda* fed on four host plants.

Parameters	Maize	Sorghum	Wheat	Rice
APOP	3.07 ± 0.11 d	3.39 ± 0.10 c	4.92 ± 0.16 a	4.17 ± 0.32 b
TPOP	31.70 ± 0.17 d	37.17 ± 0.27 c	42.92 ± 0.36 b	59.7 ± 1.08 a
Oviposition (days)	4.56 ± 0.08 a	3.87 ± 0.07 b	3.56 ± 0.07 c	2.00 ± 0.01 d
Fecundity	1088.8 ± 26.5 a	591.6 ± 11.5 b	435.6 ± 6.91 c	49.6 ± 4.54 d
*R*_0_ (offspring)	735.1 ± 59.6 a	340.2 ± 33.3 b	272.09 ± 23.9 b	7.45 ± 2.08 c
*r* (d^−1^)	0.181 ± 0.002 a	0.140 ± 0.002 b	0.117 + 0.002 c	0.032 ± 0.004 d
*λ* (d^−1^)	1.19 ± 0.002 a	1.150 ± 0.003 b	1.124 ± 0.002 c	1.032 ± 0.005 d
*T* (d)	36.4 ± 0.182 d	41.57 ± 0.279 c	47.65 ± 0.37 b	61.975 ± 1.14 a

*R*_0_ = net reproductive rate, *r* = intrinsic rate of increase, λ = finite rate of increase, *T* = mean generation time, means sharing similar letters within a row are not significantly different at *p* > 0.05.

## Data Availability

All data analyzed in this study are included in this article.
